# Negative CO_2_ emissions via enhanced silicate weathering in coastal environments

**DOI:** 10.1098/rsbl.2016.0905

**Published:** 2017-04-05

**Authors:** Filip J. R. Meysman, Francesc Montserrat

**Affiliations:** 1 Department of Analytical, Environmental and Geo-Chemistry, Vrije Universiteit Brussel, Pleinlaan 2, 1050 Brussels, Belgium; 2 Aarhus Institute of Advanced Studies (AIAS), Aarhus University, Hoegh-Guldbergs Gade 6B, 8000 Aarhus C, Denmark

**Keywords:** climate change, carbon dioxide removal, enhanced weathering, olivine, marine ecosystems

## Abstract

Negative emission technologies (NETs) target the removal of carbon dioxide (CO_2_) from the atmosphere, and are being actively investigated as a strategy to limit global warming to within the 1.5–2°C targets of the 2015 UN climate agreement. Enhanced silicate weathering (ESW) proposes to exploit the natural process of mineral weathering for the removal of CO_2_ from the atmosphere. Here, we discuss the potential of applying ESW in coastal environments as a climate change mitigation option. By deliberately introducing fast-weathering silicate minerals onto coastal sediments, alkalinity is released into the overlying waters, thus creating a coastal CO_2_ sink. Compared with other NETs, coastal ESW has the advantage that it counteracts ocean acidification, does not interfere with terrestrial land use and can be directly integrated into existing coastal management programmes with existing (dredging) technology. Yet presently, the concept is still at an early stage, and so two major research challenges relate to the efficiency and environmental impact of ESW. Dedicated experiments are needed (i) to more precisely determine the weathering rate under *in situ* conditions within the seabed and (ii) to evaluate the ecosystem impacts—both positive and negative—from the released weathering products.

## 1. Enhanced silicate weathering as a negative emission technique

To achieve the goals of the 2015 UN climate agreement, it will not be sufficient to solely reduce greenhouse gas emissions, but also CO_2_ needs to be actively captured from the atmosphere [1,2]. This has recently stimulated research into so-called negative emission technologies (NETs), sometimes also referred to as carbon dioxide removal (CDR) approaches [3]. Enhanced silicate weathering (ESW) is one of several NETs that is increasingly gaining attention as a means to deliberately remove atmospheric CO_2_ [3,4]. Compared with other NETs, ESW has the benefit that it also may counteract ocean acidification [5], which is considered an important threat to marine ecosystems [6]. The central idea behind ESW is to speed up this natural CO_2_ neutralization process by artificially increasing the weathering rate of silicate minerals [7,8]. When silicate minerals are subject to chemical weathering, a slow dissolution process is initiated, which produces alkalinity and binds CO_2_ as bicarbonate in aqueous form (table 1), thus allowing the ocean to store more CO_2_. The rate of silicate weathering can be accelerated by: (i) selectively exposing (ultra)mafic source rocks that are enriched in silicate minerals with high dissolution rates; (ii) increasing the reactive surface area—and thus the dissolution rate—by pulverizing the source rock into small particles; and (iii) distributing the mineral particles in locations with high weathering rates.

**Table 1.** The CO_2_ sequestration mechanism of olivine weathering. The dissolution of olivine consumes protons, and hence, increases the alkalinity of environment (e.g. the soil pore fluid in terrestrial soils or the pore water of marine sediments). In response, the acid–base dissociation reactions of the carbonate system in seawater will remove dissolved CO_2_, so additional CO_2_ can be taken up from the atmosphere. When secondary mineral formation reactions occur, these tend to reduce the alkalinity release by primary olivine dissolution, and so the overall CO_2_ sequestration efficiency is lower (see e.g. [9] for a more detailed discussion). At present, it is unclear how important these secondary mineral reactions are under natural conditions in the seafloor.

	primary weathering reaction
olivine dissolution	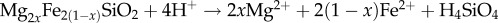
	seawater acid–base reactions
air–sea CO_2_ transfer	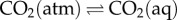
carbonic acid dissociation	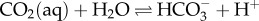
bicarbonate dissociation	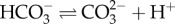
	potential secondary mineral formation
ferrous iron oxidation	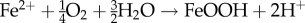
carbonate precipitation	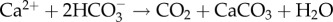
serpentinization	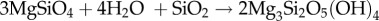
sepiolite formation	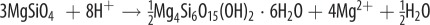

For the purpose of ESW, the mineral olivine (Mg_2(1−*x*)_Fe_2*x*_SiO_4_) has received most attention [7,8,10], as it combines a fast dissolution rate with a relative widespread abundance. Olivine dissolves three orders of magnitude faster than ordinary quartz and commercial olivine mines are operating across the globe. In terms of suitable application locations, the feasibility of enhanced olivine weathering has been evaluated for agricultural soils and tropical river basins [8,10–13], open ocean environments [14,15] and coastal zones [16]. The open ocean has the major disadvantage that olivine particles must be ground to a very small size (less than 1 µm), or else particles rapidly sink out of the surface mixed layer before being dissolved, which thus will not lead to an immediate CO_2_ uptake [15]. These small grain sizes however lead to a high energy expenditure and associated CO_2_ emissions during grinding [12], thus rendering the overall CO_2_ sequestration inefficient.

Until now, most attention has been dedicated to land-based ESW applications [8,10–13], which potentially bring a number of co-benefits in addition to CO_2_ sequestration, such as the addition of micro-nutrients, crop fertilization, soil improvement and an increased buffering of soil acidity [10,17]. The most suitable application regions for terrestrial ESW are tropical areas with high humidity, rainfall and temperature, and preferably acidic soils (i.e. low soil water pH). However, a considerable amount of this target region is covered by dense forest, and spreading vast volumes of olivine over such rainforest areas poses severe logistical challenges. Therefore, application will be restricted to arable land, and so land availability may be a limiting factor, in addition to saturation in the pore water, which has been suggested as another important limitation on the maximal CO_2_ sequestration achievable by terrestrial ESW (estimated at less than 1 Pg C yr^−1^ [13]).

The global continental shelf (28.3 × 10^6^ km^2^; [18]) is about twice the size of the total arable land surface (14.1 × 10^6^ km^2^; [19]), and so enhanced olivine weathering in the coastal zone could provide additional CO_2_ sequestration capacity. Yet until now, the potential and feasibility of applying ESW in the coastal zone remains largely unexplored [16]. Here, we address the two major research challenges for ESW in coastal systems, which essentially boil down to two simple questions: (i) ‘Will coastal ESW work as a negative emission technology?’ and (ii) ‘What are the (positive and negative) implications for coastal ecosystems?’ We will review the current knowledge on these two topics and highlight the associated uncertainties, thus providing directions for future research.

## 2. Enhanced silicate weathering in coastal systems

**Figure 1.**
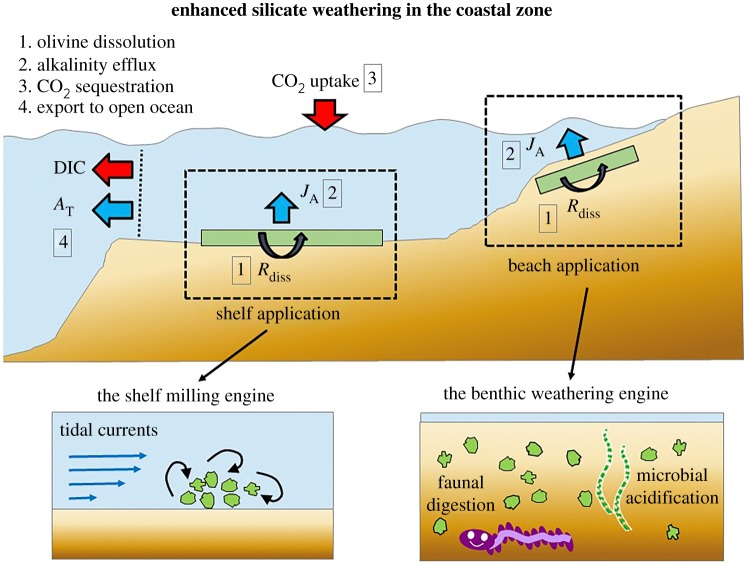
Enhanced silicate weathering in coastal systems is a four-stage process. [1] Olivine dissolution takes place at the surface of the individual mineral particles, releasing reaction products (Mg^2+^, dissolved silicate, alkalinity and trace metals) into the interstitial pore solution. [2] An additional efflux of alkalinity is released from the sediment [3]. The alkalinity increase of the surface waters induces a CO_2_ transfer across the air–sea interface. [4] Dissolution products are exchanged with the open ocean over short time scales (0.1–1 yr) and exported to the deep sea over longer time scales (100–1000 yr). Two ESW application scenarios have been proposed: (*a*) spreading coarse particles into high-dynamic shelf environments where particles are crushed during bedload transport (‘shelf milling’) and (*b*) spreading finer olivine sand onto beaches and shallows, where dissolution is enhanced through biotic processes in the seabed (‘benthic weathering engine’). *A*_T_, total alkalinity; DIC, dissolved inorganic carbon; *J*_A_, alkalinity efflux from the sediment; *R*_diss_, dissolution rate of olivine.

Two types of coastal ESW applications have been advanced (figure 1), each targeting a different depth zone of the coastal ocean. In the ‘shelf application’ scenario, one takes advantage of the fact that large areas of the continental shelf experience sufficiently high bed shear stresses capable of transporting gravel. The idea is to deposit olivine gravel (median diameter 1–5 mm) onto the seafloor in these high-energetic environments [20]. A natural grinding process occurs when sediment particles roll, hop or slide along the seabed, under the action of currents and waves. The large grain size is advantageous as one requires less energy during milling, thus generating less CO_2_ emissions during the production phase [12]. In a second ‘beach application’ scenario, fine olivine sand (median diameter 100–300 µm) is distributed in coastal areas, such as beaches and shallow subtidal areas [16]. The dissolution of olivine particles in these shallow waters could be enhanced through wave action (stimulating grain collisions) as well as through various forms of biological activity in the seabed (as discussed further below). Beach deployment could be integrated into various existing forms of coastal zone management (e.g. harbour construction works, sand nourishment on beaches).

When used as a NET, the primary goal of coastal ESW is to ensure a significant drawdown of CO_2_ from the atmosphere. The overall CO_2_ sequestration rate 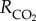
can be decomposed into two prime factors:
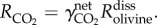


The olivine dissolution rate 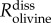 specifies how much olivine is dissolved within the seafloor per unit area per unit of time. The CO_2_ sequestration efficiency 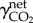 specifies the net amount of CO_2_ that is taken up from the atmosphere during the dissolution of 1 kg of olivine within the seafloor. To determine the effectiveness of coastal ESW, a prime research challenge is to achieve a better understanding of the factors that control these two parameters. More specifically, we need to reduce the uncertainty on the values that 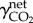 and 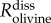 attain under *in situ* conditions, i.e. when olivine is spread in natural seafloor environments.

A number of studies have recently reviewed the CO_2_ sequestration efficiency for ESW applications [11–13,16]. These studies adopt a life cycle perspective, where one first estimates how much CO_2_ is taken up from the atmosphere by the ocean, and one subsequently subtracts any CO_2_ that is released during production, transport and application of the olivine sand used. Depending on location and application type, 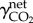 is estimated to vary between 0.5 and 1.0 ton of CO_2_ sequestered per ton of ground olivine source rock applied [11,12]. The variation in 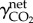 is not due to uncertainty, but results from inherent differences in the type of olivine application. The CO_2_ sequestration efficiency can differ between applications due to a number of factors, such as the geographical location (influencing the salinity, temperature and composition of the seawater, and hence the local acid–base thermodynamics and CO_2_ uptake [13,14]), the amount of inert material in the olivine source rock [5], the grain size to which the olivine rock is ground and the transport distance between the mining and application locations [11]. For a given application, these mentioned factors can be rather accurately evaluated, and so, it appears that 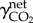 can be accurately determined upfront (say within 10% uncertainty). Still, one important uncertainty regarding the CO_2_ sequestration efficiency has only received sketchy attention: the possibility that olivine dissolution induces additional mineral formation reactions in the seabed (e.g. carbonate precipitation, clay production, sepiolite formation; table 1). These secondary reactions can substantially reduce the alkalinity released from olivine dissolution, and hence, diminish the resulting CO_2_ uptake [9]. Therefore, in order to obtain reliable predictions of the actual CO_2_ sequestration efficiency, field studies are needed in which olivine dissolution and its associated alkalinity release are assessed within the full context of sedimentary biogeochemical cycling.

The uncertainty on the olivine dissolution rate within the seafloor is even larger, as 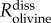 values may not even be known to within an order of magnitude. In general, the dissolution rate of olivine in or near the seabed is expressed via the kinetic rate law [15]
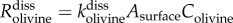


In this, *C*_olivine_ represents the amount of ground olivine distributed onto the seafloor (g m^−2^ of seabed), *A*_surface_ is the specific surface area of the mineral grains (m^2^ g^−1^), and 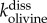 is the intrinsic dissolution rate (expressed in mol of olivine per m^2^ grain surface area per unit of time). Up until now, reliable estimates of the olivine dissolution rate under *in situ* conditions have been lacking, so model upscaling [16] has been done based on dissolution rates obtained from idealized laboratory experiments, which typically involve artificial seawater solutions free of biological activity [21–23]. These laboratory studies reveal that temperature and solution pH are the most dominant controls, where 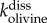 increases at higher temperatures and at lower pH, while dissolution is further promoted by the presence of organic acids [16]. Yet, an important unresolved question is how these laboratory-based kinetics relate to the actual dissolution rate of olivine in the seabed. Once olivine is spread out onto the seafloor, its chemical weathering will be influenced by a number of natural processes, which may both decrease as well as increase the intrinsic rate of olivine dissolution.

## 3. Impact of biota on weathering

Foremost, the seabed is characterized by various forms of biological activity, which could induce higher dissolution rates compared with sterile laboratory conditions. Such biological enhancement of silicate weathering has been extensively documented in terrestrial soils, giving rise to the ‘mycorrhizal weathering engine’ concept [24–26]. Mycorrhizal fungi, with their vast filamentous networks in symbiosis with the roots of most plants, can alter a large number of minerals via local acidification, targeted excretion of organic ligands, sub-micron scale biomechanical forcing and selective mobilization of cations at the mineral interface [24,25]. Although similar biologically mediated weathering processes are likely active in marine sediments, the topic has received far less attention.

Here, we propose the concept of the ‘benthic weathering engine’, where both microorganisms and invertebrate fauna act as agents of enhanced weathering in marine sediments. Together, the interplay of microbial metabolism and macrofaunal bioturbation could substantially increase the rate of olivine dissolution under *in situ* conditions. Foremost, organic matter is decomposed by a complex of microbial consortia in the seabed, which releases CO_2_ and organic acids into the pore solution. The resulting acidification of the pore water is known to stimulate the dissolution of carbonates, even when the overlying water is oversaturated, a process known as ‘metabolic dissolution’ [27]. As is the case for carbonate, metabolic dissolution will likely also stimulate olivine weathering, although the magnitude of this enhancement has not yet been quantified. One important recent finding is that porewater conditions in coastal sediments can be far more acidic than previously thought. In so-called electro-active sediments, long, filamentous microbes called ‘cable bacteria’ perform long-distance electron transport [28]. This specific metabolism induces a strong acidification (down to pH∼5) of the top few centimetres of the sediment, which greatly stimulates the dissolution of acid-sensitive minerals such as carbonates and iron sulfides [29,30]. Electro-active sediments may be globally common in the coastal zone [31], and so, these acidic marine sediments could be a target location for small-scale field trials of coastal ESW.

Additionally, large macrofauna that inhabit the sediment may also contribute to enhanced olivine weathering through the process of bioturbation [32]. In some coastal areas, like the intensely bioturbated sand flats of the southern North Sea, the entire top approximately 15 cm of sediment can pass multiple times a year through the gut of large deposit-feeders [33,34]. Gut transits at high enzymatic activity and low pH, in combination with mechanical abrasion during ingestion and digestion, have been shown to increase silicate mineral dissolution rates [35–38]. Yet the extent to which this occurs is poorly quantified, and so, an important challenge for future studies is to quantitatively determine the impact of the ‘benthic weathering engine’ on olivine weathering.

Second, olivine dissolution rates reported from laboratory studies typically describe the initial dissolution from cleansed surfaces. However, it is well known that in a later stage, secondary coatings and cation-depleted layers may evolve, which can progressively slow down the dissolution process [39]. Thus the question arises to what extent such weathering crusts will form under natural conditions. Compared with terrestrial applications, weathering crusts may be less of a problem in marine applications. In the shelf application, olivine particles are subjected to high energy conditions, where currents will cause constant grain abrasion during bedload transport, counteracting the formation of surface layers [20]. In beach applications, sediment particles will also be subject to wave motion, while additionally, mineral grains can be crushed and sheared during deposit-feeding activities of infauna. While these physical and biological transport modes will probably reduce the tendency to build up thick surface layers [16], this hypothesis still needs verification under field conditions.

Third, sediments can show saturation behaviour and consequent reduced dissolution at higher pH [40], a process that also has been advanced to limit terrestrial ESW [13]. This effect is likely not so relevant for the shelf application, where dissolution mainly occurs on top of the seabed, and so the solution surrounding the particles is continuously refreshed. However, in the beach application, olivine particles are mixed into the seabed, and upon dissolution, the alkalinity released upon olivine dissolution will tend to increase the pH of the pore water. At the same time, coastal sediments also experience physical and/or biological irrigation, which will interchange the pore water with overlying water, and hence prevent the build-up of dissolution products. In sufficiently permeable sediments, the pore water is physically flushed through advective pore flow induced by waves and currents [41]. Likewise, in sediments inhabited by burrowing macrofauna, the pore water solution is refreshed by bio-irrigation through the continuous or episodic ventilation of burrows [34,42]. Accordingly, the pore water will reflect a balance between end-product accumulation and flushing, and when end-product accumulation dominates, solubility and pH effects may put a limitation on the amount of olivine *C*_olivine_ that can be deposited per application round. The extent to which solubility and pH effects occur under *in situ* conditions is presently unknown, and should be a prime focus of future studies and controlled experiments.

## 4. Impact of weathering on biota

In addition to alkalinity, olivine dissolution releases a range of other dissolution products (silicate, Mg^2+^, trace elements) that can be transferred out of the seabed. Magnesium is not expected to pose a real concern, given its high background concentration in seawater, but the accumulation of other compounds in the overlying water could have important ecosystem effects, both positive and negative. Up until now, model studies have primarily adopted an ‘open ocean perspective’, assessing the impact of (terrestrial and open ocean) ESW on the chemical composition of the open ocean and the global marine carbon cycle [5,14,15,43]. These model simulations predict that large-scale implementation of terrestrial ESW in the tropics is capable of countering acidification throughout large parts of the tropical oceans. Aragonite is the form of calcium carbonate that is used for the synthesis of skeletal structures by reef-building corals and other marine biota. Under business-as-usual emission scenarios, the aragonite saturation state of surface waters substantially decreases, while by contrast, ESW is capable of keeping saturation state constant under rising CO_2_ levels [5]. Silicate weathering also releases dissolved silicon (DSi) and iron (Fe), which are limiting nutrients for phytoplankton in large parts of the ocean [14,15]. Accordingly, ESW could not only impact the carbon cycle through CO_2_ sequestration induced by increased alkalinity, but also by silicon and iron fertilization and stimulation of the biological carbon pump [15].

**Figure 2.**
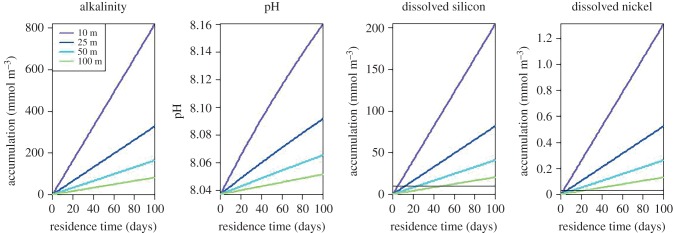
The accumulation of reaction products (alkalinity, pH, silicate, nickel) from olivine dissolution within the coastal bottom water. The accumulation 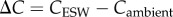 (difference in concentration with and without ESW) is plotted as a function of the water residence time for four different average water depths. For reference, the black lines indicate typical values for the ambient concentration of dissolved silicon and nickel in temperate coastal waters.

Coastal spreading of olivine, however, may induce a different geochemical cycling compared with terrestrial or open ocean applications. Foremost, when distributing olivine onto the seafloor, certain components may be selectively retained within the sediment environment. This is likely the case for iron, which forms an important trace component of olivine [9]. Under oxic conditions, ferrous iron will likely be oxidized to iron (hydr)oxides, while under anoxic conditions, ferrous iron will be trapped in the sediment as iron sulfides. Accordingly, when ESW is applied in a coastal setting, the potential for additional CO_2_ sequestration through iron fertilization seems reduced. Second, when ESW is applied in coastal systems, dissolution products may first accumulate to higher concentrations within coastal waters, before being exported away offshore and diluted into the vast open ocean (figure 1). To illustrate this, figure 2 shows the accumulation of dissolution products (alkalinity, dissolved silica, trace metals) for a typical olivine ESW application in a coastal body over a selection of water depths (10, 25, 50, 100 m) and a representative range of residence times in coastal water bodies (0–100 days). In open margins with short residence times, most products will be exported to the open ocean and the ecosystem impact will be relatively small. By contrast, in shallow continental shelf seas with long residence times, local accumulation can be important. For a shallow water system (10 m water depth) and a long residence time (100 days), the change in the carbonate system can be significant (ΔTA = 800 µmol kg^−1^; ΔpH = 0.12; figure 2), thus illustrating how ESW could also counter the effect of ocean acidification on a local or regional scale (without the need for the large-scale alteration of the chemical composition of the entire surface ocean). At the same time, the accumulation of dissolved silicon and trace metals (of which nickel is likely the most prominent) can be substantial and increase the levels of these substances way above their background level (ΔSi up to 20 times the ambient seawater concentration; ΔNi up to 45 times the ambient concentration). Although a large body of knowledge exists on marine biogeochemistry, and insights can be gained by analogy with other impacts (e.g. selective Si fertilization of coastal ecosystems by glacial meltwater), we contend that a proper assessment of the ecosystem-level impacts of ESW will necessitate a combination of mesocosm studies and large-scale field trials, given the complexity of marine ecosystem functioning.

## 5. Conclusion

If negative emissions are to become part of our future climate policies, there is a collective need to increase our knowledge base and thus to substantially accelerate our research efforts. NETs should not be regarded as a substitute for substantial emission cuts, but as complementary strategies, and could prove particularly useful to achieve the ‘last mile of decarbonization’, off-setting greenhouse gas emissions from industry, aviation and agriculture that are difficult to mitigate. Application of ESW in the coastal zone could lift the competing claims on land use imposed by terrestrial NETs, such as bio-energy with carbon capture and storage (BECCS), afforestation or terrestrial ESW [4]. Coastal ESW can be directly integrated into existing practices of coastal zone management (e.g. coastal defence and beach nourishment) and can be executed with existing technology and industrial operations (mining, shipping and coastal dredging operations).

Large-scale geoengineering is nevertheless controversial, and so societal acceptance imposes an important constraint on any future application of NETs. ESW accelerates the natural long-term fate of fossil CO_2_ [44], and in this way, it falls into the category of soft geoengineering, which may be advantageous for gaining societal acceptance (as it enhances a natural process). As coastal ESW also counteracts acidification [5], it could be part of a broader strategy for geochemical management of the coastal zone, safeguarding specific coastal ecosystems from the adverse impact of ocean acidification, such as important shellfisheries or coral reefs.

Still, as advanced here, there are two major unresolved research questions concerning coastal ESW. One critical unknown is the mineral reactivity under *in situ* conditions, which determines the time scale over which any CO_2_ sequestration can be achieved. To better constrain these dissolution kinetics, dedicated experiments are needed, investigating the biological, chemical as well as physical factors that influence olivine dissolution rates under natural conditions. Second, the large-scale release of dissolution products into the coastal environment could have important (cumulative) ecosystem impacts, both positive and negative, and should be a priority for future research. The environmental impact of coastal ESW will strongly depend on the scale of olivine application, the characteristics of the coastal water body (e.g. residence time) and the particular biota present (e.g. coral reefs will react differently compared with seagrasses). Questions on the efficacy, impact and safety of coastal ESW can only be reliably answered by suitably large field experimentation, combined with dedicated model efforts. Performing such field trials must not imply any endorsement, but should only target a critical and impartial evaluation of all direct and indirect ecosystem effects, and need to concur within the legal framework of London Protocol [45].
